# Short-term assays for mesenchymal stromal cell immunosuppression of T-lymphocytes

**DOI:** 10.3389/fimmu.2023.1225047

**Published:** 2023-09-26

**Authors:** Maryanne C. Herzig, Barbara A. Christy, Robbie K. Montgomery, Carolina Cantu-Garza, Gema D. Barrera, Ji H. Lee, Nicholas Mucha, Jennifer R. Talackine, Isaac A. Abaasah, James A. Bynum, Andrew P. Cap

**Affiliations:** ^1^ Blood and Shock Research, US Army Institute of Surgical Research, Fort Sam Houston, TX, United States; ^2^ Department of Surgery, University of Texas, Health Science Center, San Antonio, TX, United States

**Keywords:** mesenchymal stromal cell, peripheral blood mononuclear cells, phosphatidyl serine, caspase, cytokine, tNF-alpha, IL-6

## Abstract

**Introduction:**

Trauma patients are susceptible to coagulopathy and dysfunctional immune responses. Mesenchymal stromal cells (MSCs) are at the forefront of the cellular therapy revolution with profound immunomodulatory, regenerative, and therapeutic potential. Routine assays to assess immunomodulation activity examine MSC effects on proliferation of peripheral blood mononuclear cells (PBMCs) and take 3–7 days. Assays that could be done in a shorter period of time would be beneficial to allow more rapid comparison of different MSC donors. The studies presented here focused on assays for MSC suppression of mitogen-stimulated PBMC activation in time frames of 24 h or less.

**Methods:**

Three potential assays were examined—assays of apoptosis focusing on caspase activation, assays of phosphatidyl serine externalization (PS+) on PBMCs, and measurement of tumor necrosis factor alpha (TNFα) levels using rapid ELISA methods. All assays used the same initial experimental conditions: cryopreserved PBMCs from 8 to 10 pooled donors, co-culture with and without MSCs in 96-well plates, and PBMC stimulation with mitogen for 2–72 h.

**Results:**

Suppression of caspase activity in activated PBMCs by incubation with MSCs was not robust and was only significant at times after 24 h. Monitoring PS+ of live CD3+ or live CD4+/CD3+ mitogen-activated PBMCs was dose dependent, reproducible, robust, and evident at the earliest time point taken, 2 h, although no increase in the percentage of PS+ cells was seen with time. The ability of MSC in co-culture to suppress PBMC PS+ externalization compared favorably to two concomitant assays for MSC co-culture suppression of PBMC proliferation, at 72 h by ATP assay, or at 96 h by fluorescently labeled protein signal dilution. TNFα release by mitogen-activated PBMCs was dose dependent, reproducible, robust, and evident at the earliest time point taken, with accumulating signal over time. However, suppression levels with MSC co-culture was reliably seen only after 24 h.

**Discussion:**

Takeaways from these studies are as follows: (1) while early measures of PBMC activation is evident at 2–6 h, immunosuppression was only reliably detected at 24 h; (2) PS externalization at 24 h is a surrogate assay for MSC immunomodulation; and (3) rapid ELISA assay detection of TNFα release by PBMCs is a robust and sensitive assay for MSC immunomodulation at 24 h.

## Background and introduction

Mesenchymal stromal cells (MSCs) are at the forefront of the cellular therapy revolution (1–3) whether for use in graft vs. host disease (GVHD) (4, 5), as a recently approved treatment of anal fistulas (6), or as an adjunct to CAR-T cell immunotherapy (7). There were over 1,240 clinical trials for MSCs between the years 2016 and 2020 highlighting their promise (8–11). Their potential was also recently realized in several COVID therapies (12–15). The most comprehensive and up-to-date summary on the use of MSCs for COVID19 was recently published by Couto et al. in *Frontiers Immunology* 2023 (16).

MSCs are adult, stem-like cells commonly isolated from bone marrow, adipose, umbilical cord, or cord blood (17). While the source of MSC results in notable differences in tissue factor expression and subsequent hemocompatibility (18–20), all MSC preparations are currently being considered for clinical use as these cells have demonstrated immunomodulatory and regenerative properties, suggesting their profound potential in areas of tissue engineering and cellular and gene therapies (1, 2, 21–23).

The most important mechanism of action of MSCs is thought to be immunomodulation (21, 24) for mitigation of inflammatory responses (23, 25, 26). The biological responses induced by MSCs have been studied at length and include inhibition of PBMC proliferation and reduction of subsequent inflammation and pro-inflammatory cytokine release. Thus, potency assays focusing on immunomodulation have been developed to validate potential effectiveness of MSCs and MSC products for release (27–31).

*In vitro* immunomodulation assays are routinely based on the ability of MSCs or MSC-produced products to block the effects of T-cell activation (21, 23, 32) and/or T-lymphocyte proliferation (techniques reviewed by 33). Assays based on the suppression of PBMC proliferation routinely take from 4 to 7 days depending on whether a mixed lymphocyte response (7 days) or a mitogen response (4 days) is followed (34–39). Readouts of these assays range from dilution of fluorescently labeled cell constituents (e.g., using CFSE or Cell Violate Trace) to monitoring DNA replication with incorporation of bromodeoxyuridine (40) or tritiated nucleotides (41) to simple DNA quantitation by Hoechst assay (42). Surrogate markers for peripheral blood mononuclear cell (PBMC) proliferation include nuclear accumulation of Ki67 in another flow cytometry-based proliferation assay (43), increases in mitochondria with tetrazolium-based dyes in absorbance assays (44), or increases in ATP content proportional to cell number in a luminescent-based assay (45).

Shorter duration assays are based on effects on T-cell activation itself and the events that occur with activation, although these assays can also be extended to longer time points (4 days; 43). The published assays have primarily examined cytokine release, including IL1, IL6, IL10, and TNFα (46–49). Monitoring lymphocyte blastogenesis by changes in mean diameter size on an automated cell counter due to mitogen stimulation has also been proposed as a 48-h assay although data from a 12-h short-term assay were also shown (50).

In this study, we examined mitogen activation of PBMCs for opportunities to detect MSC potency at times shorter than 72 h. Here, we report on MSC potency assays of immunomodulation examined by markers of apoptosis, caspase assays, and phosphatidyl serine externalization (PS+), as well as PBMC production of tumor necrosis factor alpha (TNFα). These assays use either flow cytometry or rapid enzyme-linked immunoassay (ELISA) methods and cryopreserved pooled donor human PBMCs in co-culture with MSCs to determine suppressive ability. Results are compared to current standard methods—either a 72-h proliferation assay by ATP measurement or a 96-h assay of proliferation by CFSE label dilution. Both PS externalization and TNFα release prove to be robust assays of MSC potency.

## Materials and methods

### Cellular preparations—MSCs

Human MSCs were purchased from RoosterBio, Inc. (Frederick, MD), expanded in the recommended medium, and cryopreserved at the initial passage following receipt. Population doublings were limited to less than 15–20.

### PBMCs

Whole blood was collected from healthy donors in citrate tubes according to an approved institutional standard operating procedure. PBMCs were purified using gradient centrifugation through Ficoll-Paque Premium (1.078 g/mL density, VWR, Lutterworth, Leicestershire, England) in Greiner Bio-One Leucosep tubes (Cat # 227290P, Greiner Bio-One North America Inc., Monroe, NC) as described by the manufacturer (45). Briefly, 50 mL of blood per donor was collected in citrated tubes by the USAISR Research Blood Bank; PBMCs were separated from plasma and red blood cells and washed twice with Dulbecco’s phosphate buffered saline (dPBS, Gibco #14190-144, without calcium or magnesium chloride). Both single donor (hSD-PBMC) and pooled donor (hPD-PBMC, 8–10 donors) PBMC preparations were prepared. PBMCs were resuspended at 1–2 × 10^7^ per mL in CryoStor10, frozen at 1°C/min cooling rate in a Nalgene™ Mr Frosty and stored in liquid nitrogen until use. Viability upon thaw was 94 ± 2.4% (*n* = 9) by 1:1 dilution with acridine orange propidium iodide (AOPI) stain and cell counting in the Cellaca MX High-Throughput Automated Cell Counter (Nexcelom Bioscience, Lawrence, MA).

### PBMC stimulus and proliferation

Cryopreserved PBMCs were rapidly thawed; washed twice in PBMC media (RPMI without phenol red, Cat # R7509, Sigma Aldrich) containing 10% MSC qualified FBS (cat # 12662011, Thermo-Fisher), 1 mM Glutamax (Cat # 35050061, Thermo Fisher), and 10 mM HEPES, pH 7.4 (Cat # H0887, Sigma Aldrich); and resuspended at 1.5 × 10^6^ PBMCs per mL in PBMC media. In 96-well plate assays (Corning 96-well flat bottomed, Tissue culture treated, polystyrene plates, Sigma Aldrich Cat # CLS3595), 150,000 PBMCs were loaded per well with or without MSC co-culture in a volume of 200 µL. Proliferation was stimulated by the addition of 50 µL of stimulus as indicated in the text. Stimuli included phytohemagglutinin P (PHA-P, Sigma Aldrich Cat # L-1668) and phytohemagglutinin-L (PHA-L, Sigma Aldrich Cat # 431784). After 2, 4, 6, 24, 48, 72, or 96 h incubation at 37°C, 5% CO_2_, cells in the media were resuspended and aliquots were taken for analysis. In designated experiments, a monocyte-depleted population of PBMCs was obtained by incubating the thawed, washed, resuspended PBMCs at 1.5 × 10^6^ in culture media in a T-75 tissue culture flask overnight. After incubation, monocyte-depleted PBMCs were removed, washed in culture media, and resuspended at 1.5 × 10^6^ cells per mL for use in experiments. After overnight incubation, monocyte-depleted PBMCs were generally 85% viable.

### MSC co-culture experiments

For routine experiments, MSCs were serially diluted starting at 50–60,000 cells/well and cultured in 96-well plates for 24 h (45). On Day 0, the attached MSCs were washed, re-fed with PBMC media, and incubated with or without 150,000 freshly thawed pooled PBMCs/well, in the presence or absence of stimulus. Actual PBMC : MSC ratios ranged from 2.5:1 to 202.5:1. In selected experiments, “licensing” of the MSCs was performed by incubating the cultured MSCs for the 72 h prior to harvest in 50 μg/mL interferon-gamma (IFN-γ).

### Proliferation measured by CFSE label dilution using flow cytometry

The classic carboxyfluorescein succinimidyl ester (CFSE) assay was performed in 96-well plates as previously described (36, 45). MSC co-culture experiments were performed as above with PBMCs labeled with 5(6)-carboxyfluorescein diacetate N-succinimidyl ester (CFDA, Sigma #21888-25mg-F, St Louis, MO), converting CFDA to CFSE. After incubation with or without MSCs in the presence or absence of mitogen, at 37°C for 96 h, labeled PBMCs were assayed by flow cytometry. Cells were stained with anti-CD3 antibody, and to determine the lymphocyte population, uptake of the nucleic acid dye 7-aminoactinomycin D (7AAD; BioLegend Cat # 420404) discriminated live/dead staining. Specifically, cells were centrifuged at 400 × *g* for 6 min, resuspended in 50 µL of Hanks’ Buffered Saline Solution (HBSS) containing 2% bovine serum albumin (BSA) and then incubated with 20 µL of APC-CD3 antibody (BD Biosciences Cat# 555342, RRID : AB 398592) for 20 min at room temperature. After using the wash-only setting on the BD FACS Lyse Wash assistant, 5 µL of 7AAD was added and the cells were incubated for an additional 5 min before analysis on a BD FACS Canto II flow cytometer (BD Biosciences, Franklin Lakes, NJ) using an expanded FSC-A/SSC-A gate to capture the larger proliferating population (51). A total of 20,000 CD3+ events were captured when possible. Proliferation of PBMCs was shown by the dilution of CD3+/CFSE+ signal with diminished signal intensity (36, 45, 52, 53). The CFSE data were calculated as % proliferated of viable CD3+ population. Suppression was calculated as described previously (45) as 1 − ((Sample − UnstimControl)/(StimControl − UnstimControl)), where UnstimControl are unstimulated PBMCs without MSCs and StimControl is the value of mitogen-stimulated PBMCs without MSCs. In some calculations, MaxSignal, the highest value determined, was used instead of StimControl when the lowest cell numbers of MSCs in co-culture experiment synergized the PBMC immune response.

### Proliferation measured by luminescent ATP assay

Proliferation of both MSCs and PBMCs was assessed by using ATP levels as a surrogate for cell number and quantitation of ATP levels using the bioluminescent reagent Cell Titer-Glo 2.0 (Promega Cat # G9242, Madison, WI) as described previously (45, 54). At 72 h, unless specified elsewhere, non-adherent PBMCs were resuspended with gentle trituration and 50 µL was were mixed with 50 µL of Cell Titer-Glo, 2.0 in a white Lumitrac plate (Greiner Bio-One Cat # 655075). Growth or attrition in the co-cultured MSCs was monitored by removal of suspension PBMCs, washing with 250 µL of dPBS, addition of 75 µL of dPBS to each well, followed by 75 µL of Cell Titer-Glo. After 30 min, 100 µL of the MSC samples was transferred to the white Lumitrac plate for assay. Luminescent signal was determined in an appropriate plate reader. The Perkin Elmer Victor3, 1420 multilabel counter, 1 s read, with Wallec software (Perkin Elmer, Waltham, MA), a Molecular Devices Spectramax M5, luminometry setting 5 repeats (Molecular Devices, San Jose, CA), and a GloMax Discover, quick read at 0.3 s integration (Promega, Madison, WI) were used interchangeably. A four-parameter logistic analysis of luminescence from contemporaneous ATP standards was used to determine ATP concentrations in the samples. Suppression was calculated as above.

### Apoptosis induction

Cryopreserved hPD-PBMCs were rapidly thawed, washed twice in PBMC media, and resuspended at 1.5 × 10^6^ PBMC per mL of PBMC media as above. In a 6-well plate, 4.5 × 10^6^ PBMCs in 2.5 mL were placed per well and treated as control (no addition), mitogen stimulated (15 µg/mL PHA-P with the addition of 7.5 µL of 5 mg/mL PHA-P) or apoptosis-induced (1 µM staurosporine with the addition of 2.5 µL of 1 mM staurosporine in DMSO; Sigma Aldrich Cat # S6942). At 24 and 48 h, PBMCs were resuspended and analyzed for viability, phosphatidylserine externalization, and caspase 3/7 activity.

### Phosphatidylserine externalization—Annexin V, Annexin V/lactadherin, and lactadherin

After treatment as above, 0.6 × 10^6^ PBMC per tube were centrifuged at 400 × *g* for 6 min and the pellet was resuspended in 100 µL of Annexin V staining buffer (BioLegend Cat # 422201, San Diego CA) or 100 µL of HBSS (for lactadherin only samples) containing 5 µL of Human TruStain FcX™ (BioLegend Cat # 422302). After a 5-min room temperature incubation in the dark, the cells were then stained with 2.5 µL of Pacific Blue™ anti-human CD45 Antibody (BioLegend Cat#304029 RRID : AB_2174123, San Diego, CA) and 5 µL of Alexa Fluor® 647 Annexin V (BioLegend Cat # 640912, San Diego, CA) with and without 1 µL of FITC-bovine lactadherin (Prolytix, Essence Junction, VT) for 15 min at room temperature in the dark. The addition of 5 µL of 7AAD for a further 5 min in the dark was used to discriminate live from dead cells. Prior to analysis, the samples were diluted with 100 µL of HBSS (Lactadherin only) or 100 µL of Annexin V buffer (Annexin V ± lactadherin samples) and then analyzed on a BD FACS Canto II flow cytometer (BD Biosciences, Franklin Lakes, NJ). Alternatively, doubly labeled cells were analyzed on an AMNIS imaging flow cytometer (Luminex, Austin, TX). Data are expressed as percent of parent.

### Phosphatidylserine externalization—lactadherin

PBMCs with or without mitogen treatment and MSC co-culture were harvested and pooled from the 96-well plates at the times specified; 1 × 10^6^ cells were centrifuged at 600 × *g* for 6 min, resuspended in 100 µL of HBSS containing 5 µL of Human TruStain FcX™, and incubated for 5 min at room temperature. PBMCs were then stained with the following markers: 2.5 µL of APC/Cyanine7 anti-human CD3 Antibody (BioLegend cat # 300318, RRID : AB_314054, San Diego, CA) and 2 µL of bovine lactadherin (Prolytix Cat# BLAC-FITC, Essence Junction, VT) with or without 2.5 µL of Brilliant Violet 421™ anti-human CD4 antibody (BioLegend Cat #357424, RRID : AB_2721519, San Diego, CA). Cells were stained for 15 min at room temperature in the dark followed by a 5-min incubation using 5 µL of 7AAD (BioLegend, San Diego, CA). HBSS (100 µL) was added to the samples before analysis on a BD FACS Canto II flow cytometer (BD Biosciences, Franklin Lakes, NJ). Data are expressed as % positive cells of the live population. Suppression was calculated as above.

### Caspase analysis—fluorescently labeled cells

Caspase 3/7 activity was determined by automated cell counting. After incubation at 37°C for 2, 4, 6, 24, 48, or 72 h, control, mitogen-stimulated, and apoptosis-induced samples were resuspended, aliquoted into 0.6 mL of polypropylene micro-centrifuge tubes and mixed with 12.5 µL of a 1/50 dilution of NucView™ caspase 3/7 reagent in the ViaStain^TM−^Cell Fitness Panel (Nexcelom Cat # CSK-V0024-1) for a further 30-min incubation at 37°C before transfer to a Cellaca MX plate and collection of brightfield and fluorescent images on a Cellaca MX automated cell counter using the caspase 3/7 apoptosis assay. Caspase active cells are expressed as percent of total cell number determined by brightfield analysis.

### Caspase analysis—enzyme activity on luminescent substrate

Activity of caspases 3/7, 8, and 9 were determined from singly cultured or co-cultured PBMCs and MSCs incubated in the presence or absence of mitogen as described above. At either 24 or 72 h, PBMCs in each well were triturated to suspend the PBMCs in media and 25-µL aliquots were diluted into 25 µL of dPBS in a white LUMITRAC assay plate; 50 µL of the appropriate caspase assay reagent was added (Promega Caspase-Glo 3/7, Cat # G8091; Caspase-Glo 8, Cat # G8201; and Caspase-Glo 9, Cat # G8211). After 30–60 min, luminescent readings were determined in a dedicated luminometer. Data are expressed as relative luminescent readings (RLU).

### Cytokine analysis—ELISA

Tumor necrosis factor-alpha, interleukin 6, and interleukin 10 (TNFα, IL6, and IL10, respectively) were quantitated by multiplexed ELISA using the Ella Simple Plex platform from Bio-Techne (Minneapolis, MN) according to manufacturer’s recommendations. Conditioned media from stimulated or unstimulated PBMSC-MSC cocultures treated as described above in 96-well plates were frozen, thawed on the date of assay, and centrifuged at 20,000 × *g* for 5 min before analysis. Samples were diluted threefold in ELISA kit assay buffer; 60 µL from each sample was added to the dedicated ELISA plate and the plate loaded onto the automated ELISA. Cytokine levels were determined after 70 min. Alternatively, a rapid, no-wash ELISA (Lumit TNFα ELISA, Promega) was performed, which relies on proximity interactions of bound antibody to reconstitute a light-generating enzyme that can produce luminescence signals. Samples were either from frozen, thawed, centrifuged media from mitogen-stimulated assays or on samples taken directly from mitogen-stimulated samples without centrifugation and were assayed according to the manufacturer’s recommendations. For thawed frozen samples, 50 µL each of diluted standards or centrifuged media was added to a 96-well Lumitrac plate followed by the addition of 50 µL of a 2× antibody mix and then 60-min room temperature incubation, per the manufacturer’s recommendations. For freshly harvested samples, 80 µL of diluted standards or resuspended PBMCs was transferred to a 96-well Lumitrac plate with 20 µL of 5× antibody mix, per the manufacturer’s recommendations. After incubation, 25 µL of room temperature equilibrated, diluted detection substrate in buffer was added to each well. After brief mixing, and a 3- to 5-min incubation, the luminescence was read on a luminometer. The concentration of TNFα was determined from the linear standard curve. Suppression of the PBMC signal in the presence of MSCs was calculated as described above.

### RT-PCR

RT-PCR was performed as described previously (54). mRNA was isolated with the RNeasy Mini Kit (Qiagen, Germantown, MD) from washed MSCs after co-culture with PBMCs. cDNA was generated using the RT2 HT First strand kit (Qiagen). mRNA expression of target IDO and the housekeeping gene B2M was quantified by Real-Time PCR (RT-PCR) using validated mRNA primers (Quantitect Primer Assay, Qiagen), cDNA, and the i-Taq Universal SYBR Green Supermix (BioRad, Hercules, CA) on a PTC Tempo Thermal Cycler (BioRad) performed in 96-well clear multi-plates. Specific IDO mRNA expression was normalized to B2M gene expression.

### Statistics

ATP standards were fit to a four-parameter dose–response logistic curve using GraphPad Prism version 8.1.2 for Windows, GraphPad Software, Inc., San Diego, California USA (www.graphpad.com). TNFα standards were calculated internally (Simple Ella platform) or were determined by linear regression using GraphPad Prism, version 8.1.2 for Windows. Significant differences were determined with one- and two-way ANOVA using JMP version 15.2.0, from SAS Institute, Inc., Cary, NC, USA (www.jmp.com). Pairwise comparisons of means were made using Tukey HSD. Significance was set at *p* < 0.5. Unless otherwise noted, data are presented as average ±standard deviation.

## Results

### Apoptosis of PBMC as a potential metric of immunosuppression

When PBMCs undergo stimulation, the proportion of CD4+/CD3+ PBMCs decreases as CD8+/CD3+ increase (54–57). There are reports that MSCs induce apoptosis in co-culture (58). To determine if the CD3+ or CD3+/CD4+ population undergoing apoptosis might be a useful marker for immunomodulation by MSCs, we examined traditional markers of apoptosis especially for time frames earlier than 72–96 h (59, 60).

### Measurement of “late apoptosis” of PBMC through caspase 3/7 activity

**Figure 1:**
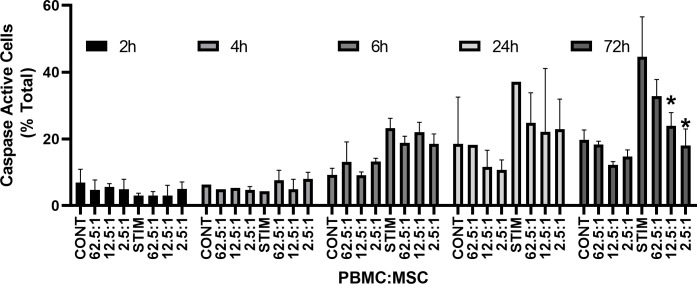
Apoptosis. MSC co-culture effects on Caspase 3/7 activity. Control and 15 µg/ml PHA-P-stimulated hPD PBMCs are shown at 2, 4, 6, 24 and 72 h. MSC co-culture at the listed ratios are shown for both unstimulated and stimulated samples. Averages ± std dev are shown. Significance is set to *p* < 0.05 and samples significantly different from stimulated control are shown with an *.

Caspase activation can be determined using NucView, a cell-permeant DNA binding dye with a linked DEVD peptide sequence (Asp-Glu-Val-Asp). Cells with caspase activity cleave the DEVD peptide, allowing the dye to bind DNA and display bright green fluorescence. In validating the assay, cells were monitored at 24, 48, and 72 h using an automated cell counter (**Supplementary Figure 1A**). Caspase 3/7 activity is evident 24 h after treatment with 15 µg/mL PHA-P, but not to the level of cells treated with staurosporine (Control, 5.5% ± 1.4%; PHA-P, 17.8% ± 3.6%; staurosporine, 26.5% ± 3.8%). Along with the observed increase in caspase activity, there is a concomitant decrease in viability (**Supplementary Figure 1B**) (Control, 86% ± 3.0%; PHA-P, 63% ± 7.2%; staurosporine, 34.4% ± 6.9%). By 48 h, caspase activity in PHA-stimulated PBMCs is stable (Control, 8.9% ± 1.14%; PHA-P, 21.2% ± 3.4%; staurosporine, 59.3% ± 7.4%) and viability has actually increased. In contrast, virtually all the cells are now dead after treatment with the known apoptosis inducer staurosporine (Control, 83.0% ± 1.6%; PHA-P, 72.3% ± 5.4%; staurosporine, 1.5% ± 2.14%). There is both a dose and time dependence for caspase 3/7 activity in PHA-stimulated PBMCs (**Supplementary Figure 1C**). Having established the validity of this assay in measuring caspase 3/7 activity on PBMCs, the effect of MSCs on PBMCs caspase 3/7 activity was determined. PBMCs were co-cultured with and without MSCs, with and without PHA stimulation. Caspase 3/7 activity by NucView labeling was determined at 2, 4, 6, 24, and 72 h after initiating co-culture. At 2 and 4 h, no significant difference with PHA stimulation is seen; however, by 6 h and for 24 h, PHA-stimulated PBMCs are significantly different from control PBMCs (*p* < 0.0001 and *p* = 0.001, respectively), although there was no significant difference with MSC co-culture at these times. There was significant immunosuppression of PBMC caspase activity by MSC co-culture only at the 72-h time points (**Figure 1**).

### Measurement of “late apoptosis” using multiple luminescent caspase assays

**Figure 2:**
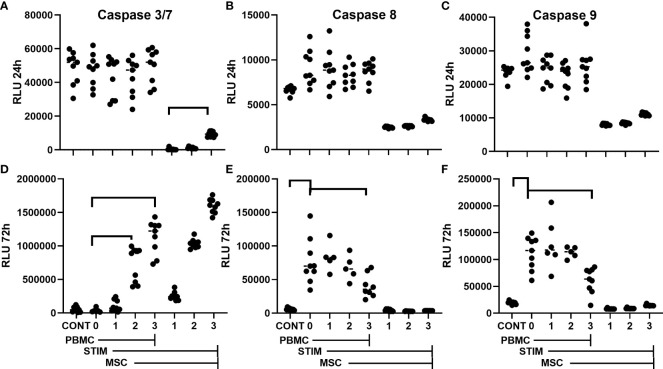
Apoptosis-caspase luminescent assay. Caspase activity in hPD PBMC and MSC with and without co-culture. PBMCs were at 150,000 per well; MSCs listed as 0, 1, 2, or 3 were at 0, 2,400, 12,000, or 60,000 cells/well. All except control were stimulated with 15 µg/ml PHA-P and then analyzed for caspase activity by luminescent assay. **(A–C)** are samples analyzed at 24 h; **(D–F)** are samples analyzed at 72 h. **(A, D)** are Caspase 3/7 activity; **(B, E)** are Caspase 8 activity; **(C, F)** are Caspase 9 activity. Data are aggregated from one BM-MSC co-cultured with three different PBMC donor preparations. PBMC samples significantly different from stimulated controls by Tukey’s determination are designated. Significance is set at *p* < 0.05. Bars indicate significant differences from control in co-culture experiments.

Caspase 3/7 is only one family of caspases. Therefore, luminescent-based assays were used to detect activity by other caspases. These assays also rely on the cleavage of caspase specific tetrapeptides (DEVD, caspase 3/7; LEHD, caspase 9; LETD, caspase 8). In these assays, aminoluciferin becomes the substrate for a proprietary thermostable luciferase upon cleavage of the respective tetrapeptide, generating light that is detected with a luminometer. Using these luminescent assays for caspase activity, caspase activity of stimulated PBMCs (15 µg/mL PHA-P) was examined. Triplicate determinations for caspase 3/7, caspase 8, and caspase 9 were run on each sample. Data from three experiments are shown. These experiments were done in co-culture in the presence or absence of three different concentrations of one hBM-MSC preparation that had been allowed to attach for 24 h prior to co-culture with PBMCs to examine the ability of MSC co-culture to suppress caspase activation. Controls included PBMCs in the absence of stimulus and PHA-treated MSCs without PBMCs. Caspase activity was assayed on PBMC samples at 24 and 72 h (**Figure 2**). In comparison to the previous method, caspase 3/7 showed little activation in stimulated PBMCs at 24 h (**Figure 2A**). Caspase 8 and caspase 9 do show slight increases in activity with stimulation (*p* = 0.0392 and *p* = 0.0758, respectively), but hBM-MSCs in co-culture show little suppression of PBMC caspase activity (**Figures 2B, C**). By 72 h after stimulation, PBMC caspase 3/7 levels are indistinguishable from those in unstimulated cells (**Figure 2D**). In this assay, caspase 3/7 activity is only evident when MSCs are present, and it is apparent that MSCs contribute the bulk of the signal, as seen in MSCs cultured alone (**Figure 2D**). However, for both caspase 8 and caspase 9 activity (**Figures 2E, F**), a significant, dose-dependent suppression of caspase activity is seen with MSC co-culture. MSCs co-cultured at the highest concentration significantly suppress caspase activity relative to that seen in PBMCs cultured alone (*p* < 0.001 for both caspase 8 and 9). While this suggests that monitoring caspase activity could be used in potency assays, the 72-h time frame does not markedly improve other existing potency assays following PBMC stimulation.

### Measurement of “early apoptosis” via PS externalization

Phosphatidylserine (PS) externalization is commonly referred to as a marker of early apoptosis (60–63). However, PS externalization does not necessarily always lead to apoptosis and can instead be a hallmark of cellular shape change (64–66). Upon mitogenic stimulation, PBMCs undergo a “blast” change, accompanied by an increase in side scatter by flow cytometry (**Supplementary Figure 2**) (55). Monitoring this blast size change has been suggested as a potency assay for MSCs (50). PS staining allows easy discrimination of this shape change. For this assay, we monitored PBMC PS externalization by lactadherin binding. While annexin V staining is the most commonly used stain for PS+, its calcium binding requirement can be problematic under some conditions. Lactadherin is a multifunctional secreted extracellular matrix glycoprotein with a stereo-specific binding affinity (67) for PS+ surfaces. It has a lower threshold affinity for PS+ binding than annexin V (68–71) and, as illustrated in **Supplementary Figure 2A**, shows more extensive binding to PHA-stimulated PBMCs compared to annexin V, although apoptosis-induced, staurosporine-treated cells show similar levels of annexin V and lactadherin binding. Lactadherin’s pattern of staining is similar to annexin V (**Figure 2B**). Live cells without PS binding show no annexin V binding; live cells staining with lactadherin and not annexin V show a diffuse PS+ staining pattern, while live cells staining with annexin V show similar staining with lactadherin.

### PS+ gating strategy

The flow cytometry gating strategy for PS externalization is shown in **Supplementary Figure 3**. For these studies, the FSA-A/SSC-A gate is widened to detect stimulated lymphocytes (**Supplementary Figure 3A**). Unstimulated (**Supplementary Figure 3A**) or stimulated (**Supplementary Figure 3C**) PBMCs at various time points are stained with fluorescent dyes and antibodies to distinguish live and dead cells, CD3+ cells, CD4+ cells, and PS+ cells with fluorescently labeled bovine lactadherin. With this gating strategy, a dose dependence for PS detection can be seen using two different forms of phytohemagglutinin—PHA-P, with two molecular forms containing both leucoagglutinin and erethroagglutinin activity, and PHA-L, which is the purified phytohemagglutinin-leukocyte reactive subunit. These two mitogens have different sensitivities, and lactadherin staining of PS+ cells shows this dose dependence. **Supplementary Figure 4** also shows results of different gating strategies. Gating for both CD4+/CD3+ parent cells does not markedly increase sensitivity of PS+ staining over CD3+ parent cells alone. **Supplementary Figures 4A–D** demonstrates that this staining does not progress or change over time following treatment. The percentage of PS+ cells at 6 h and 24 h are not significantly different, whether analyzing the entire CD3+ population or the CD4+/CD3+ population subset. This is further illustrated in **Supplementary Figure 4E** with data from multiple different hPD PBMC preps. **Supplementary Figure 4E** shows average PS+/CD3+ live cells at 2, 4, 6, 24 and 72 h. At all time points, there is a significant increase in the PS+ population of CD3+ live cells with mitogen stimulation.

### Monitoring PS externalization by flow cytometry for an MSC potency assay

**Figure 3:**
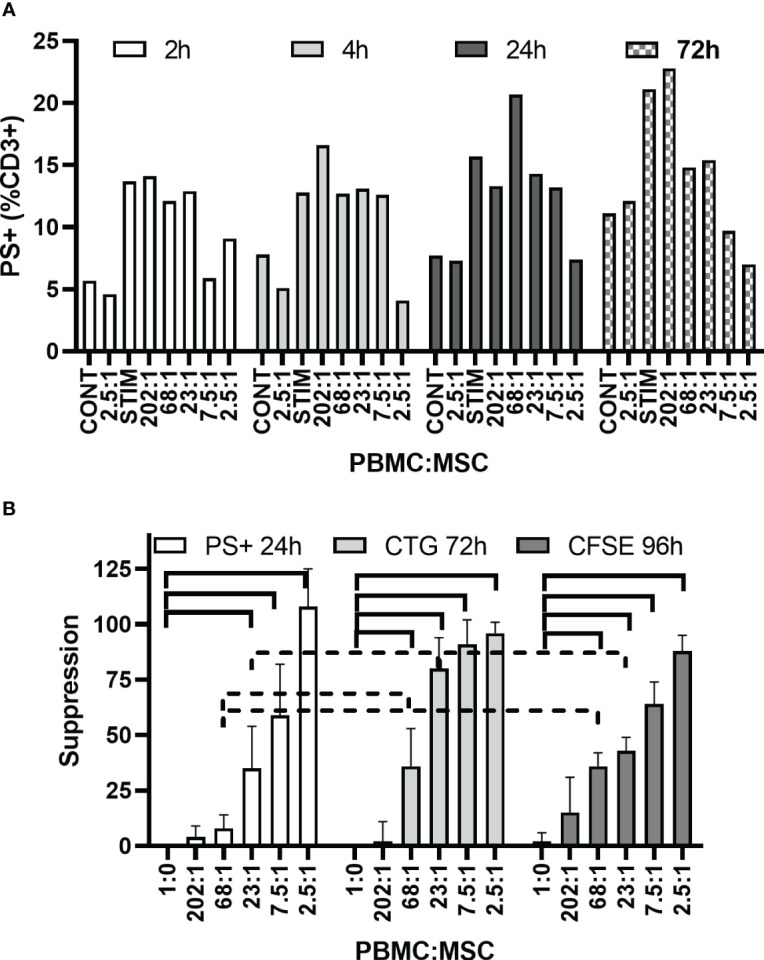
PS externalization. Time dependence for effects of MSC co-culture. **(A)** Representative experiment shows hPD PBMC and BM-MSC co-culture over time. hPD PBMCs were incubated with BM-MSCs allowed to attach for 24 h prior to PBMC addition. Ratios of hPD PBMCs to BM-MSCs are listed below the graph. Stimulation was with 15 µg/ml PHA-P and samples were assayed at 2, 4, 24, and 72 h by LACTAD binding with CD3+ gating. **(B)** Assay Comparison. The aggregated data are from four different hBM-MSC preparations, one each of freshly harvested cells and freshly thawed cells from the same passage number of two different hBM-MSC sources. MSCs were allowed to attach for 24 h prior to PBMC addition and stimulation with 15 µg/ml PHA-P. The experiments were repeated three times with each sample being analyzed at 24 h for PS+ staining of the CD3+ cells, at 72 h for proliferation as analyzed by ATP, and at 96 h for proliferation by dilution of CFSE signal. Averaged data are expressed as % suppression ± std dev. Data significantly different from no MSC stimulated control are shown by solid lines; data significantly different from the other assay are shown by dashed lines (*p* < 0.05).

**Figure 3A** illustrates one representative MSC potency test by PS+ staining examined at 2, 4, 24, and 72 h. Levels of stimulated signal do not markedly increase from 2 to 24 h, with only a minor increase at 72 h. BM-MSC co-culture at the highest MSC concentrations relative to PBMC number diminishes PS+ staining, suggesting immunosuppression by the MSCs. Finally, when data from multiple experiments is expressed as PBMC suppression to allow comparison across different assay methods (**Figure 3B**), the BM-MSC suppression of PBMC PS+ signal at 24 h is remarkably similar to both a proliferation assay monitored by ATP at 72 h and by the dilution of CFSE signal in proliferating cells. By two-way ANOVA, there is a difference between these assays that does interact with the MSC concentration; however, these differences are only seen in the mid-range MSC concentrations. No differences exist at the highest MSC concentrations (PBMC : MSC ratios of 2.5:1). The PS+ staining assay most closely resembles the CFSE dilution assay.

### Monitoring MSC effects on stimulated PBMC release of TNFα

**Figure 4:**
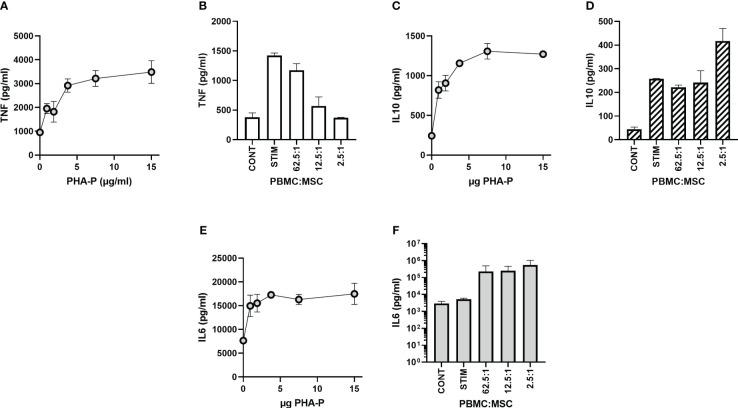
Cytokine release. **(A, C, E)** Dose dependence for TNFα, IL10, and IL6. Duplicate experiments show a dose response for cytokine release by Simple Ella multiplex ELISA. **(A)** TNFα response, **(C)** IL10 response, and **(E)** IL6 response. Stimulation was with 15 µg/ml PHA-P and samples were assayed at 24 h. **(B, D, F)** MSC co-culture effects on cytokine release. hPD PBMCs were incubated with BM-MSCs allowed to attach for 24 h prior to PBMC addition. Ratios of hPD PBMCs to BM-MSCs are listed below the graph. Cytokine levels, averages ± std dev, of triplicate experiments are shown. Note the change to log values on the *y* axis for IL6.

PBMCs respond to mitogens with multiple cytokine responses (43, 54, 72–75). For example, co-culture of PBMCs with MSCs results in augmented IL6 cytokine release, increased IL10 release, and decreased TNFα release (54). The time frames commonly assayed range from 3 to 5 days. In a multiplex assay with TNFα, IL6, and IL10, both cytokines show a dose response in PBMCs alone at 24 h, with increasing stimuli resulting in increasing signal (**Figures 4A, C, E**). However, the response to MSC co-culture differs in that PBMCs respond to MSC co-culture with TNFα, a clear-cut decrease in signal (**Figure 4B**); PBMCs increase the secretion of IL10 in the presence of MSCs (**Figure 4D**); and IL6 secretion into the media is complicated by the strong synergy seen with PBMC-MSC co-culture even in the absence of mitogen (**Figure 4F**; **Supplementary Figures 5, 6**). For a potency assay involving IL6 and IL10, it is difficult to develop metrics when there does not appear to be a ceiling. TNFα response is the most straightforward cytokine response, and Chinnadurai et al. (43) found it to have the highest correlation to proliferation. Therefore, we focused on secretion of this cytokine into the media. Here, we assayed the level of TNFα secreted by PBMCs at 2, 4, 6, and 24 h following mitogen stimulation, used this in an MSC potency assay, and compared the results to a mitogen proliferation assay at 72 h. To determine TNFα concentrations, two separate rapid ELISA platforms were used; the Simple Ella automated ELISA platform from BioTechne and a luminescent, no wash ELISA from Promega.

### Time and dose dependence

In **Supplementary Figure 7**, both time and dose dependence of TNFα in the media were determined by Simple Ella ELISA in the absence of MSC co-culture. Serial dilutions of either PHA-L or PHA-P were used as mitogens for hPD PBMC preparation. There is an expected dose response, and unlike the results for PS externalization, there is also a change in response over time.

### Monitoring TNFα levels as a potency assay

**Figure 5:**
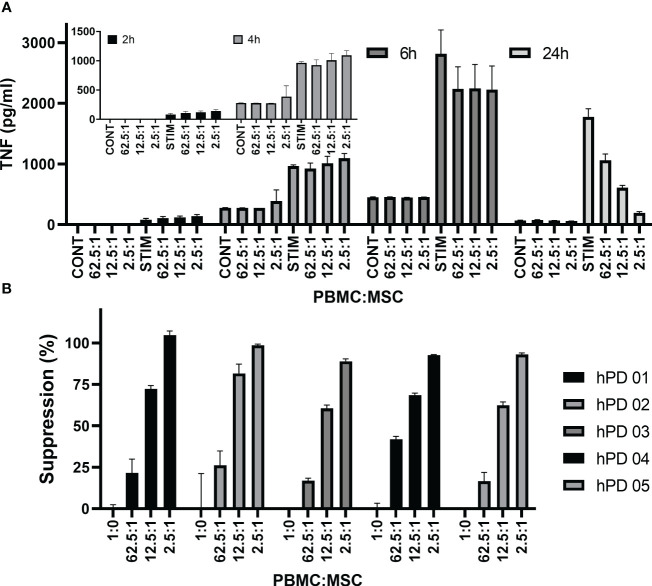
TNFα cytokine assay. Time dependence of MSC co-culture. **(A)** Representative experiment shows hPD PBMC/BM-MSC co-culture. hPD PBMCs were incubated with BM-MSCs allowed to attach for 24 h prior to co-culture. Ratios of hPD PBMCs to BM-MSCs are listed below the graph. Stimulation was with 15 µg/ml PHA-P and conditioned media samples were assayed at 2, 4, 24, and 72 h by luminescent TNF-a ELISA assay. **(B)** Reproducibility of assay with multiple hPD PBMC preparations. Suppression of TNFα secretion was calculated for one BM-MSC preparation co-cultured with multiple hPD PBMC preparations. Averages ± std dev of three independent samples per experiment are shown. TNFα levels were determined by luminescent ELISA.

**Figure 6:**
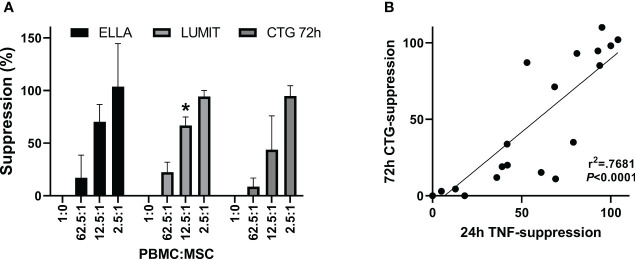
TNFα cytokine assay. Comparison of suppression. determined by the two assay methods. **(A)** Aggregate data from one BM-MSC preparation assayed for 24 h TNFα secretion by automated ELISA, ELLA (*n* = 5), or by luminescent ELISA, Lumit (*n* = 7) and for 72-h proliferation by ATP measurements, CTG (*n* = 5). Five different hPD PBMC preparations and two different hSD PBMC preparations were used in the aggregated data. Two-way ANOVA showed no difference for assay*concentration (*p* = 0.22) with the PBMC : MSC ratio treatment being significant (CONC *p* < 0.0001). Values significantly different from CTG assay shown by *. **(B)** Correlation of data from panel **(A)**.

**Figure 5A** shows TNFα concentrations from a representative PBMC : MSC co-culture experiment at 2, 4, 6, and 24 h. At early times (2–6 h after stimulation), low levels of TNFα are detected and co-culture with MSCs has little effect even at the highest relative level of MSCs (2.5:1: PBMC : MSC). A dose-responsive suppression of secreted TNFα by stimulated PBMCs is pronounced by 24 h. This signal is independent of hPD PBMC preparation. In **Figure 5B**, suppression data from multiple experiments performed in triplicate using five different hPD PBMCs show the same response for TNFα release at 24 h. This assay is robust and reproducible with an averaged intra-experimental error of 3.5%, an inter-experimental variation of 10.9%, and an average 27 fold increase in signal from control to stimulated values. The aggregated data for one BM-MSC preparation assayed both by TNFα release (at 24 h) and by PBMC proliferation (at 72 h) are graphed in **Figure 6A**. Both automated ELISA and a luminescent ELISA assay were compared with a 72-h proliferation assay utilizing ATP values of PBMCs. By two-way ANOVA, while there is the expected significant difference for PBMC : MSC ratio (*p* < 0.0001), no difference exists between assay type (*p* = 0.2369). A correlation of *r* ^2^= 0.74 is found (**Figure 6B**).

**Figure 7:**
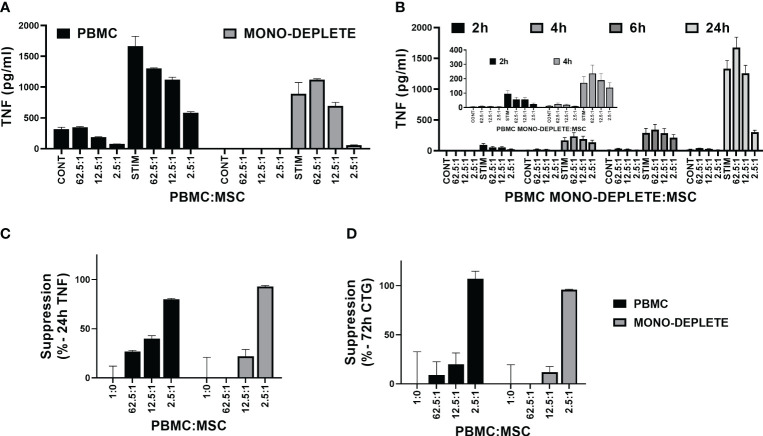
Effect of monocyte depletion of PBMCs on TNFα cytokine assay. Comparison of MSC co-culture with PBMC. **(A)** Representative experiment shows hPD PBMC (solid bar) or monocyte-depleted hPD PBMC (gray bar) in co-culture with BM-MSC co-culture. hPD PBMCs were incubated with BM-MSCs allowed to attach for 24 h prior to co-culture. Ratios of hPD PBMC populations to BM-MSCs are listed below the graph. Samples were treated with or without 15 µg/ml PHA-P for 24 h before assay of conditioned media samples by automated ELISA. **(B)** Time dependence of MSC co-culture on TNFα secretion by monocyte-depleted PBMC. Representative experiment shows monocyte-depleted hPD PBMCs incubated with BM-MSCs allowed to attach for 24 h prior to co-culture. Ratios of hPD PBMCs to BM-MSCs are listed below the graph. Stimulation was with 15 µg/ml PHA-P and conditioned media samples were assayed at 2, 4, 24, and 72 h by automated TNFα ELISA assay. Inset graph highlights background values seen at 2 and 4 h. Comparison of suppression of cytokine stimulation of hPD PBMC and monocyte depleted PBMCs. **(C)** Suppression of TNFα secretion seen in panel **(A)** was calculated for both hPD PBMC (solid bar) and monocyte-depleted PBMCs (gray bar). Averages ± std dev of three independent readings per sample are shown. Comparison of suppression of hPD PBMC and monocyte-depleted PBMCs at 72 h proliferation. **(D)** Representative experiment shown in **(A)** was allowed to progress to 72 h before determination of proliferation by luminescent ATP assay. ATP levels were determined in triplicate for hPD PBMCs (solid bar) and monocyte-depleted PBMCs (gray bar).

Ribeiro et al. (76) had developed an assay for LPS-driven TNFα production by monocytes. Therefore, we investigated whether the TNFα signal was derived from monocytes in the PBMCs. We performed a simple monocyte depletion of the PBMCs by incubating them overnight on tissue culture plastic, allowing the monocytes to attach. The next day, the remaining PBMCs were removed from the flask, washed, and compared in co-culture experiments with PBMCs freshly thawed and washed from cryopreservation. **Figures 7A–D** show the results of a representative experiment, performed in triplicate. Overnight incubation to monocyte depleted the PBMCs and lowered the initial background levels of TNFα secretion to near zero (**Figure 7A**). The time course from 2 to 24 h shows a similar pattern to non-monocyte-depleted PBMCs (**Figure 7B** vs. **Figure 5A**) and both 24-h suppression by TNFα secretion and 72-h suppression by ATP assay are similar (**Figures 7C, D**, respectively). Thus, overnight incubation to deplete monocytes results in a PBMC population with an increase in TNFα secretion of ~260-fold above baseline vs. freshly thawed PBMCs with an increase in TNFα secretion above baseline of ~3.4 fold; the ultimate signal of TNFα secretion at 24 h for the monocyte-depleted cells is ~71% of the hPD PBMC preparations, and immunosuppression values show no significant difference between the two populations (average of two experiments performed in triplicate).

## Discussion

Potency assays are critical to the cellular therapy field for initial analysis, FDA approval, and release criteria or to potentially enable point-of-care decisions. The ability of MSCs to modulate activated PBMCs is a linchpin of many MSC potency assays. This study reexamined the mitogen activation of PBMCs hoping to exploit some characteristics that occur in a shorter time frame than the normal 3–7 days for the evaluation of proliferation suppression.

PBMCs have a series of programmed responses to mitogen; for example, CD69 and CD154 positivity on the cell surface increase in CD3+ T-lymphocytes by 24 h (46, 77) and could be suppressed by human regulatory T cells (Treg) (46). However, CD69 surface expression appears insensitive to MSC suppression (54). CD3+ T-lymphocytes consist of both helper CD4+ and cytotoxic CD8+ cells. As the response to stimulus progresses, the proportion of CD4+/CD3+ cells decreases and CD8+/CD3+ cells increase (54, 56, 57); these changes are blocked by MSC co-culture.

There is conflicting information in the literature about the apoptotic consequences of mitogen activation of PBMCs with and without MSC co-culture. While lymphocyte apoptosis was found to be inhibited by co-culture with MSCs (78, 79), Plumas et al. reported that MSCs induce apoptosis in activated lymphocytes (80). Meanwhile, Karaoz et al. (58) monitored Annexin V binding and concluded that MSC co-culture induced apoptosis, but only after 4 days of co-culture.

The advent of rapid assays for activated caspases that do not require cumbersome purification and flow cytometric procedures suggested that, if present, investigating apoptosis might be a fruitful avenue for improved potency assay development. In response to this, we hypothesized that monitoring apoptosis might be a robust assay for immunosuppression at time points earlier than 72–96 h.

To investigate apoptosis, both a classic “early apoptosis” marker (binding to externalized phosphatidyl serine residues) and “late stage” apoptosis markers (caspase activity) were used. The apoptotic response generally completes within 6–24 h of stimulation, so even the late-stage apoptosis becomes a possible candidate for a rapid immunomodulation assay. While caspase 3/7 activity was evidence of late-stage apoptosis and was seen in mitogen-activated PBMCs, reliable suppression of this activity by MSC co-culture only occurred at 72 h. Thus, this assay did not allow for earlier assessment of MSC suppression activity.

Luminescent assays for individual caspases in addition to caspase 3/7 were used to examine whether BM-MSC in co-culture with PBMCs could modulate any apoptotic response. Caspase 3/7, 8, and 9 activity was detected at 24 h, but no significant increase was seen upon mitogen stimulation with *in situ* luminescent assays. In this assay, there was no caspase 3/7 activity detected with MSC co-culture that could be attributed to PBMC activation; all caspase 3/7 activity in this assay appeared to be due to the plated MSCs. This contrasts with the significant caspase 3/7 activity detected at 24 h on PBMCs alone treated with PHA and staurosporine in the previous assay. The reason for this discrepancy is unclear. Possibly the release of caspase 3/7 by MSCs alone masked PBMC activity. Caspase 8 and 9 activity in mitogen-stimulated PBMCs was detected at 72 h and that activity was suppressed by MSC co-culture. However, while suppression of caspase 8 and 9 activation in mitogen-stimulated PBMCs might be a surrogate for immunomodulation by MSCs, the time frame is such that it provides no improvement over current assays. Whether this activity is due to apoptosis is in question; there are reports of non-apoptotic caspase 8 and caspase 9 activity (81–84).

Whether PS externalization detected here by Annexin V binding is an indicator of early apoptosis or is non-apoptotic is unclear. PS externalization is a hallmark of apoptosis (61–63, 85), yet there are clear reports of the uncoupling of PS externalization from apoptosis (86–88) and previous observations suggested that PS externalization is non-apoptotic for PBMCs (89, 90). PS externalization could also simply reflect the cell surface changes seen in blastogenesis (88). A shape change or blast event has long been known for PBMCs (50, 91–93); in order to monitor proliferation by flow cytometry, one needs to widen the PBMC gate. It is logical to assume that with membrane remodeling comes redistribution of phophatidylserine to the cell surface (54).

For studies investigating the potency assay potential of PS+ assessment, another known phosphatidyl serine binding protein was used, bovine lactadherin (69, 71). Lactadherin has simplified buffer requirements compared to Annexin V, and it has a linear binding response rather than the threshold response that is seen for annexin V (69). Examining either CD3+ or CD4+/CD3+ cells after mitogen activation, a dose-dependent increase in PS+ binding was seen. Even at the earliest time point measured of 2 h, a significant increase in PS+ labeling was seen after mitogen stimulation; however, there was little time-dependent increase in PS+ binding after 2 h, suggesting that there is no accumulation of cells with PS+ surfaces. As has been noted before, the lowest concentrations of MSCs (relative to PBMCs) are counterproductive, having a stimulating effect rather than suppression in these assays (35, 54, 94–97). Complete suppression is only reliably seen at PBMC-to-MSC ratios of 2.5:1, representing 150,000 non-adherent PBMCs to 60,000 plated MSCs, which, in a volume/size consideration, gives the advantage to the MSCs. Using PS externalization as a marker, we were able to show a similar level of suppression of PBMC proliferation by MSCs whether in the CFSE dilution assay after 96 h, an ATP assay of proliferation after 72 h, or suppression of PS externalization at 24 h.

The final aspect of mitogen activation of PBMCs considered in these studies was cytokine release. Cytokine release is a hallmark of activated lymphocytes (98–100). Most potency assays focus on the 72- or 96-h time points (43); however, TNFα secretion can be detected after 6 h of stimulation for monocytes (76). Both of these reports used flow cytometry for determination of TNFα levels. Flow cytometry can be a lengthy process, requiring expensive equipment and trained and dedicated staff. Rather than using flow cytometry for cytokine analysis, we focused on the rapid determination of cytokine levels using ELISA. The negatives of ELISA assays mostly focus on potential errors in washing or setup and the lengthy incubations that may be needed. However, the two commercial ELISA methods examined in this study were rapid, required minimal manipulation, and delivered data within 90 min of assay initiation. This means that rapid testing could conceivably be done in 1 day.

The level of TNFα was chosen as the surrogate marker for immunomodulation, as TNFα levels secreted by PBMCs into the media have been previously shown to be dose dependently decreased with MSC co-culture (43, 54, 101). Both a mitogen dose dependence and a time dependence were found for PBMC secretion of TNFα. Using this method to determine the suppressive potency of MSCs at 24 h shows a strong correlation with a 72-h suppression seen in the proliferation assay with an *r* ^2^ of 0.74. The sensitivity, rapidity, and ease of the TNFα assay illuminate the promise of this method.

IL6 was also investigated and revealed a complex interplay of PBMCs and MSCs in the presence or absence of mitogen. The synergy of interaction leads to orders of magnitude changes by 6 h but the lack of a ceiling limits this cytokine for a potency assay. It is a good candidate to investigate early time points and interactions in future studies.

One caveat is that this assay did not detect any cryolesions using two separate BM-MSC preparations (**Supplementary Figure 8**). The cryolesion of freeze-thawed samples is known and alternative storage methods are investigated (39, 45, 102–106). Cryolesions possibly result from the bi-directional interaction of PBMCs and MSCs (54, 96). Quality of freezing, thawing, and inherent cell properties may impact the cryolesion in immunosuppression assays. It is known that by 72 h in immunosuppression co-culture assays, the lower concentrations of MSCs are prone to attack by PBMCs (35, 54, 96). As cryolesions may likely be an issue of delayed MSC proliferation, it is perhaps unsurprising that a cryolesion was not detected at this 24-h time point as opposed to a 72- or 96-h time point.

The exact mechanism for the modulation by MSCs of PBMC cytokine release is unknown. Indoleamine 2,3 dioxygenase plays a role in MSC’s immunosuppression (56, 107–109). While pre-treating MSCs with IFN-γ did result in elevated IDO levels compared to non-licensed cells, it did not provide appreciable benefit to modulating PBMC cytokine secretion levels at 24 h (**Supplementary Figure 9**).

The gold standard for MSC potency characterization prior to MSC release still lies in the combinatorial matrix approach of Chinnadurai et al. (43, 54). The short-term assays here fit in with potential rapid assays for initial characterizations or for testing prior to patient use. Ribeiro et al. (76) used monocyte activation with TNFα release also in the 6-h time frame for a clinically relevant marker of MSC suppression. Gibson et al. (50) proposed an assay based on blastogenesis and mean diameter size change in PBMCs; the percent blast change increased over time with 48 h being the sweet spot for determinations.

The assays presented here are novel in the cell markers they follow, the agents used, and the assay instrumentation. They have the potential to serve industry for rapid screens of MSC potency prior to full analysis. They also could be used in laboratories with more limited resources since most will have access to a plate reader with luminescent capabilities. Purification of T cells might improve robustness of the assays; a simple overnight incubation to deplete the PBMC preparation of monocytes resulted in a robust signal-to-noise ratio increase from 3.4-fold to 2,660-fold; however, the end result of immunosuppression was not significantly different.

The intent was to find assays that might be exploited for point-of-care analysis at earlier time frames of 4–6 h to determine possible individualized patient response. Importantly, while signals were evident even at early time points for caspase activity (4 h), PS externalization (2–6 h), and TNFα secretion (2–6 h), immunosuppression by MSC co-culture could not be detected at these times for any of the methods. Immunosuppression was only reliably found at 24 h.

The promises of cellular therapies are beginning to be realized. Therapies focused on preventing secondary damage post injury and suppressing a dysregulated immune response will serve to improve patient outcomes. Although treating coagulopathic patients with pro-coagulant MSCs must be approached with caution (16, 19, 20, 103), MSCs’ immunomodulation potential suggests that their use in any population such as trauma patients who are susceptible to a hyperactive immune response and coagulopathy (110) may be beneficial. It is hoped that these short-term assays will aid in developing, screening, and customizing cell therapy for the aid of the patient.

## Author’s note

The views expressed in this article are those of the author(s) and do not reflect the official policy or position of the U.S. Army Medical Department, Department of the Army, DoD, or the U.S. Government. This study was conducted under a protocol reviewed and approved as exempt from IRB review by the US Army Institute of Surgical Research Regulatory Compliance Division and in accordance with the approved protocol.

## Data availability statement

The raw data supporting the conclusions of this article will be made available by the authors, without undue reservation.

## Ethics statement

This study used commercial mesenchymal stem cells and prepared PBMCs from an institutional blood bank. The US Army Institute of Surgical Research (USAISR) Research Regulatory Compliance Division (RRCD) did not require the study to be reviewed or approved by an ethics committee because it does not involve living individuals about whom an investigator conducting research obtains information or biospecimens through intervention or interaction with the individual, and uses studies or analyzes the information or biospecimens or obtains, uses, studies, analyzes, or generates identifiable private information or identifiable biospecimens.

## Author contributions

MH, BC, JB and AC contributed to conception and design of the study. MH, RM, CC-G, GB, JL, NM, JT, and IA performed assays related to the study. MH performed statistical analysis and wrote the first draft of the manuscript. All authors contributed to manuscript revision, read and approved the submitted version.
