# Supplementary material for: Short-term assays for mesenchymal stromal cell immunosuppression of T-lymphocytes
Source: Front Immunol. 2023 Sep 26;14:1225047. doi: 10.3389/fimmu.2023.1225047 (PMC10562633; doi:10.3389/fimmu.2023.1225047)
Supplement: Supplementary file 1 [file DataSheet_1.pdf]

Supplementary Material

Short Term Assays for Mesenchymal Stromal Cell Immunosuppression of T-Lymphocytes

Maryanne C. Herzig[1*], Barbara A. Christy[1], Robbie K. Montogomery[1#a], Carolina Cantu-Garza[1], Gema D. Barrera[1], Ji Lee[1], Nicholas Mucha[1#b], Jennifer Talackine[1], Isaac A. Abaasah[1], James A Bynum[1,2], Andrew P. Cap[1]

*** Correspondence:**

Maryanne Herzig

maryanne.c.herzig.ctr@health.mil

<u>LIST OF SUPPLEMENTAL FIGURES</u>.

Supplemental Figure 1. Apoptosis. Caspase 3/7 Activity.

Supplemental Figure 2. PS externalization. Validation of lactadherin binding assay

Supplemental Figure 3. PS externalization. Gating Strategy

Supplemental Figure 4. PS externalization. Dose and Time Dependence for CD4+/CD3+ sub-population compared with all CD3+cells.

Supplemental Figure 5. IL6 cytokine release. Time Dependence of MSC co-culture.

Supplemental Figure 6. IL6 cytokine assay. Data from Supplemental Figure 6 is re-graphed to include IL6 secretion of BM-MSCs alone with or without PHA-P stimulation.

Supplemental Figure 7. TNFα cytokine assay. Time and Dose Dependence.

Supplemental Figure 8. TNFα cytokine secretion from hPD PBMCs incubated with BM-MSCs from cultured cells or cells that had been recently thawed.

Supplemental Figure 9. Effect of 'licensed' MSCs on hPD PBMC immunosuppression.

Supplemental Figure 1.

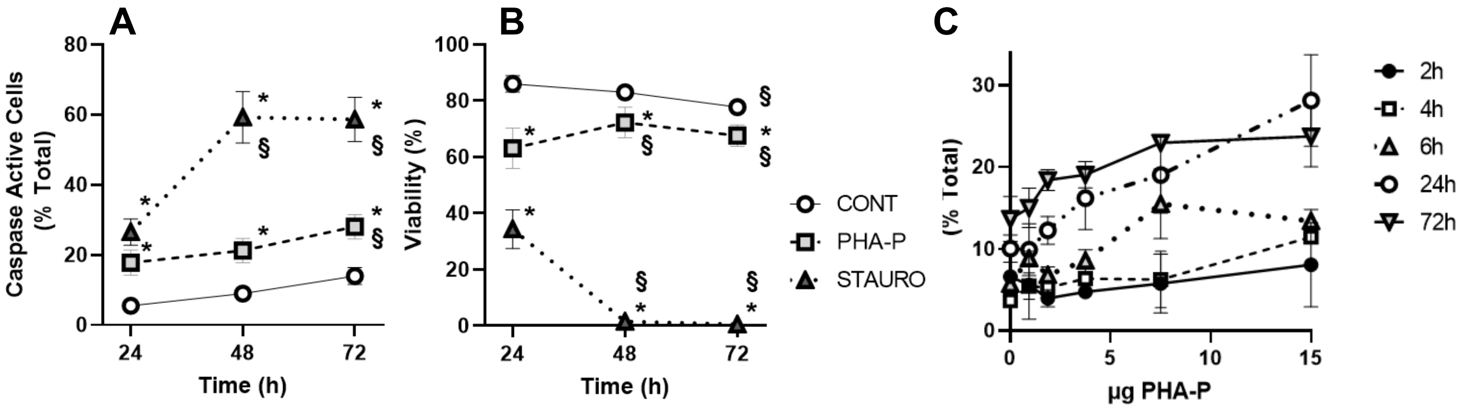

Supplemental Figure 1. Apoptosis. Caspase 3/7 Activity. (A) Time dependence of caspase activation. Caspase 3/7 positive cells of hPD PBMC at 24, 48 and 72 hr treated with 15 µg/ml PHA-P, 1 µM staurosporine or the untreated controls, were determined by automated cell counter. Averages from quadruplicate determinations in two experiments is shown. **(B) Viability** was determined by AOPI counting on an automated cell counter. Significant is set at $P<0.05$; significant differences from CONT within time point is shown by *; significant differences from 24h within treatment is shown by **§**. **(C). Dose and Time Dependence for Caspase 3/7 activity.** hPD PBMCs were stimulated with 0-15 µg/ml PHA-P for 2,4,6,24 and 72h before assay by automated cell counter. Averaged data ± std dev is from two experiments with the same hPD PBMC preparation.

Supplemental Figure 2.

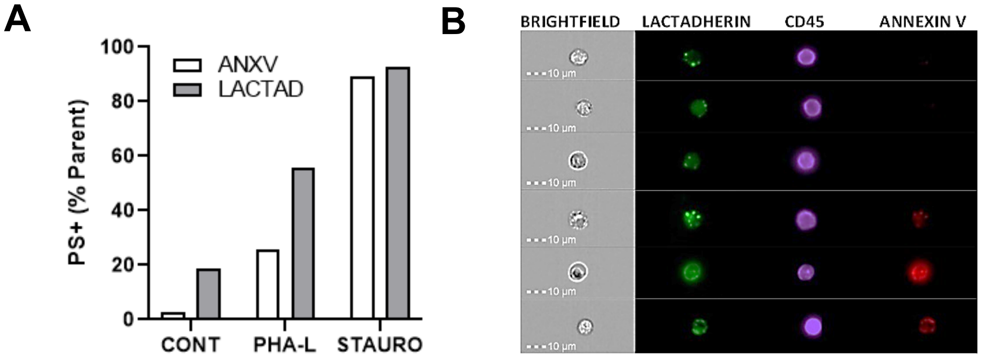

Supplemental Figure 2. PS externalization. Validation of lactadherin binding assay. (A). Direct comparison of cells stained with either annexin V (open bars) or lactadherin (grey bars) 24h after stimulation with 15µg/ml PHA-P. Data is represented as the percentage of the parent CD45+ population that is positive for binding of either lactadherin or Annexin V. **(B). AMNIS images** of hPD PBMC from Figure 3A stained with ANX V and lactadherin. Three images each of live cells with LACTAD staining, no ANX V and of live cells with LACTAD and ANX V staining. Representative images chosen from over 75000 taken.

Supplemental Figure 3.

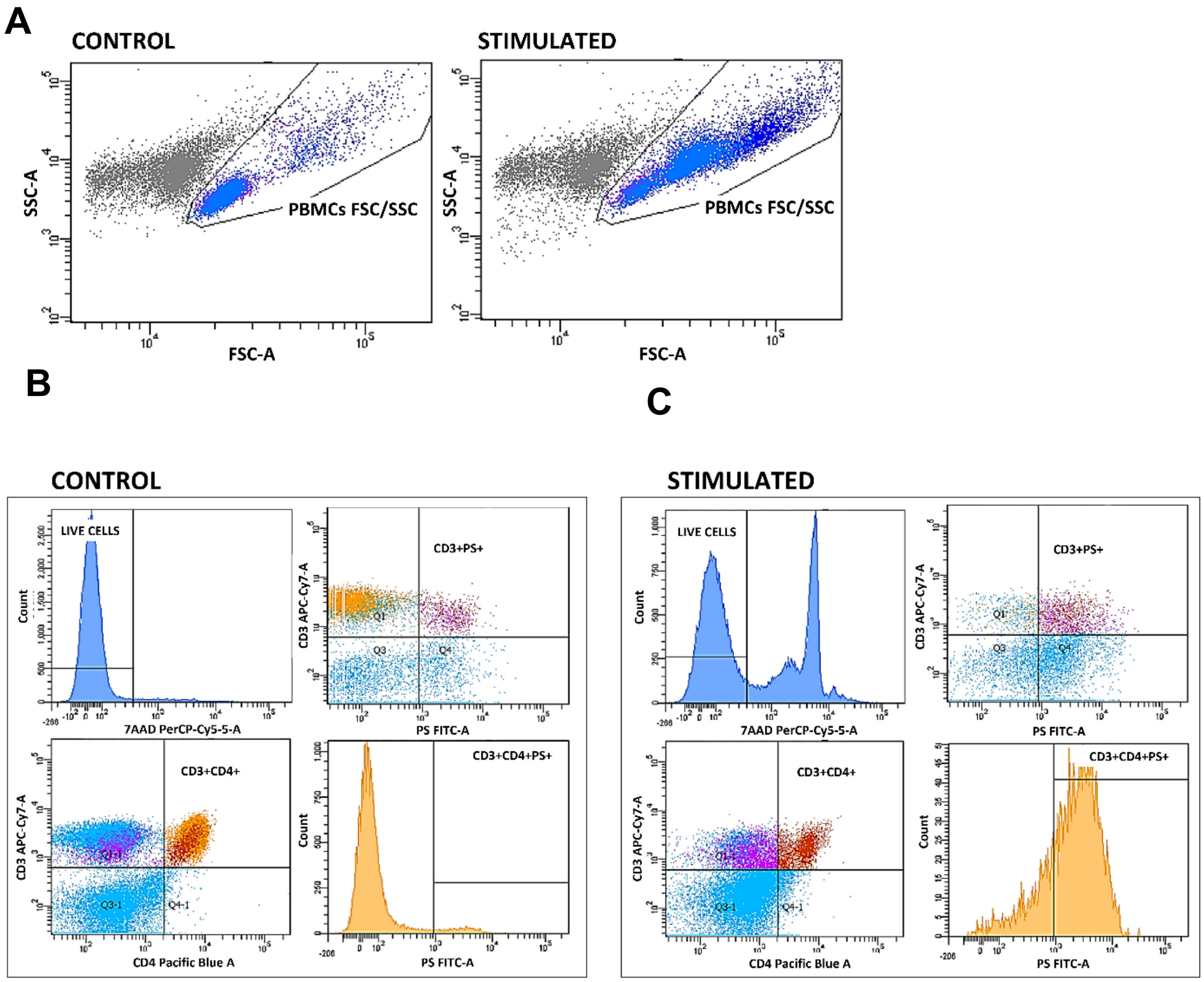

Supplemental Figure 3. PS externalization. Gating Strategy. (A). Blast shape change demonstrated by change in pattern of forward and side-scatter of PBMCs upon stimulation with 15 µg/ml PHA-P. **Flow Cytometry Gating Strategy. (B)** Gating strategy to obtain PS+ (% parent) cells is shown on unstimulated hPD PBMCs. **(C)** Flow Cytometry Gating Strategy for Stimulated PBMCs. Gating strategy is shown for 15 µg/ml PHA-P stimulated hPD PBMCs**.**

Supplemental Figure 4.

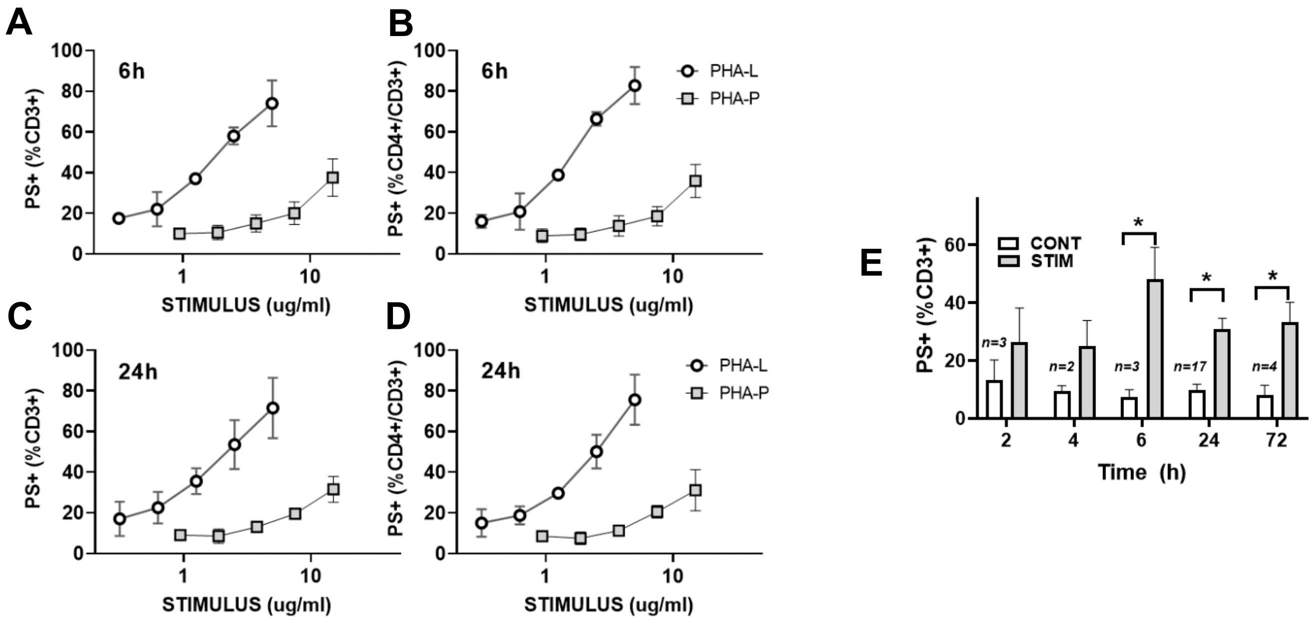

Supplemental Figure 4. PS externalization. Dose and Time Dependence for CD4+/CD3+ sub-population compared with all CD3+cells. hPD PBMCs were stimulated with PHA-L or PHA-P for 6h **(A,B)** and 24h **(C,D)** before being analyzed for %PS+ cells within the CD3+ **(A,C)** or CD+/CD3+ populations **(B,D).** Data is from duplicate experiments with the same hPD PBMC preparation. **(E)** Time dependence for PS binding in CD3+ live cells. Data is average PS+ values ± std dev for control and 15µg PHA-P stimulated PBMC preparations. Number of determinations is listed on the graph and constitutes data from 5 different pooled donor preparations.

Supplemental Figure 5.

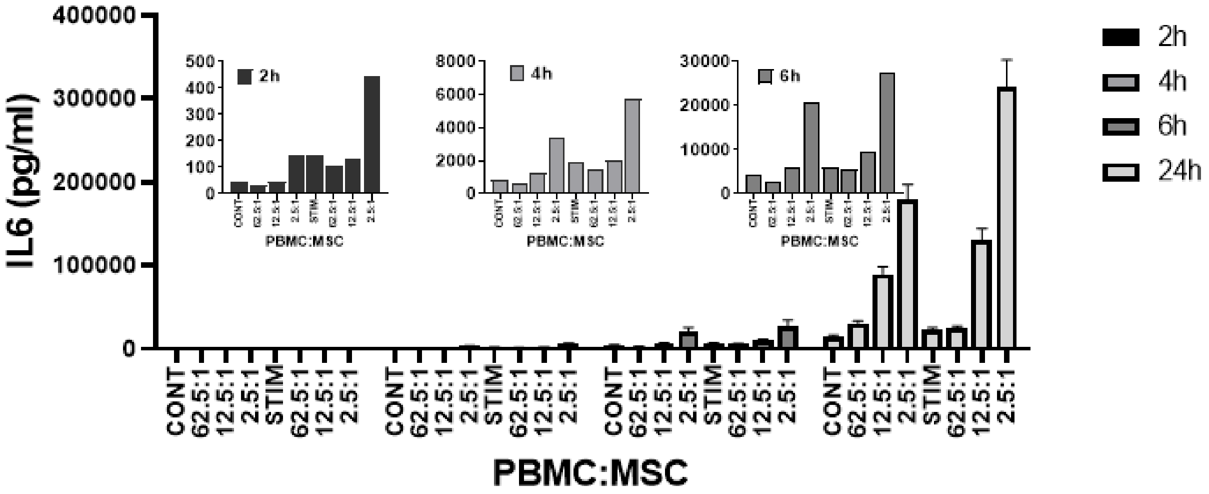

Supplemental Figure 5. IL6 cytokine release. Time Dependence of MSC co-culture. (A). Representative experiment shows hPD PBMC/ BM-MSC co-culture. hPD PBMCs were incubated with BM-MSCs allowed to attach for 24h prior to co-culture. Ratios of hPD PBMCs to BM-MSCs are listed below the graph. Stimulation was with 15 µg/ml PHA-P and conditioned media samples were assayed at 2, 4, 24 and 72 h by automated IL6 ELISA assay.

Supplemental Figure 6.

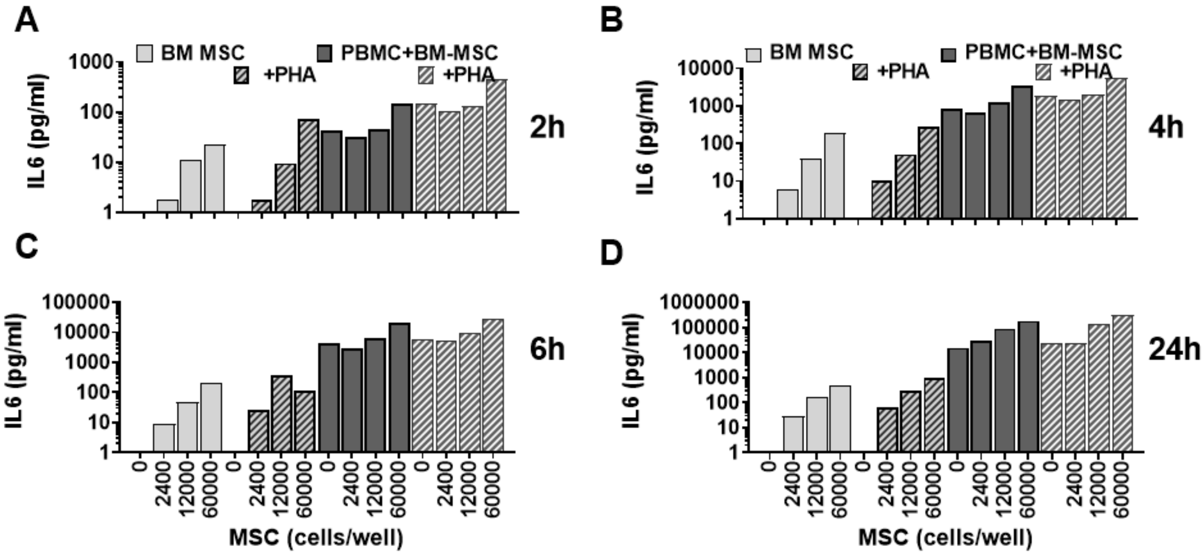

Supplemental Figure 6. IL6 cytokine release. Data from Supplemental Figure 6 is re-graphed to include IL6 secretion of BM-MSCs alone with or without PHA-P stimulation. (A), (B), (C), and **(D)** graphs are of IL6 cytokine values at 2, 4, 6 and 24 h, respectively. Ratios of hPD PBMCs to BM-MSCs are listed below the graph. Note the shift to log scale values for IL 6.

Supplemental Figure 7.

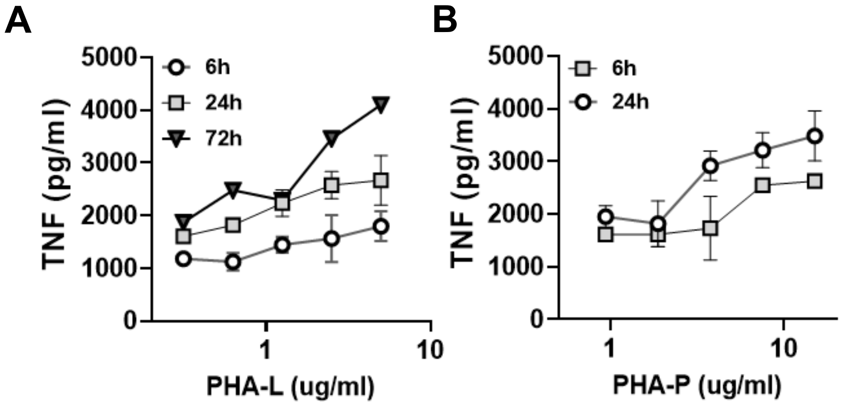

Supplemental Figure 7. TNFα cytokine assay. Time and Dose Dependence. hPD PBMCs were stimulated with increasing doses of PHA-L **(A)** or PHA-P **(B)** for 6, 24 and 72h before being analyzed for TNFα secreted into the medium by automated ELISA. Data is from duplicate experiments with 1 hPD PBMC preparation.

Supplemental Figure 8.

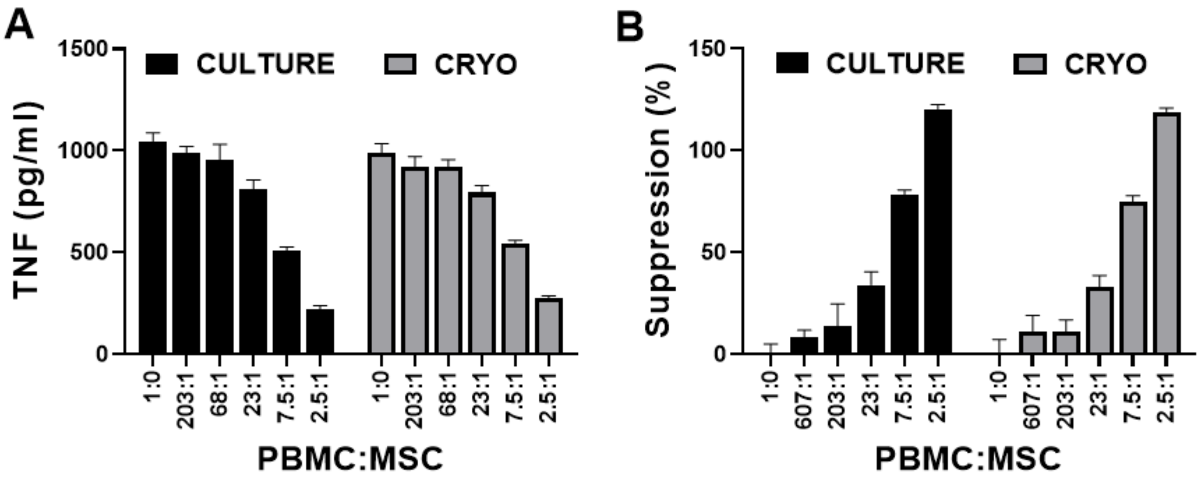

Supplemental Figure 8. TNFα cytokine secretion from hPD PBMCs incubated with BM-MSCs from cultured cells or cells that had been recently thawed. Representative experiment from one BM-MSC preparation performed in triplicate is shown. Averaged data is from luminescent TNFα ELISA. BM MSC cells from P6 culture or freshly thawed from p5 cryopreservation were allowed to attach for 24h (culture= black bar; cryopreserved= grey bar). Panel **(A)** is TNFα levels; Panel **(B)** is calculated suppression values.

Supplemental Figure 9.

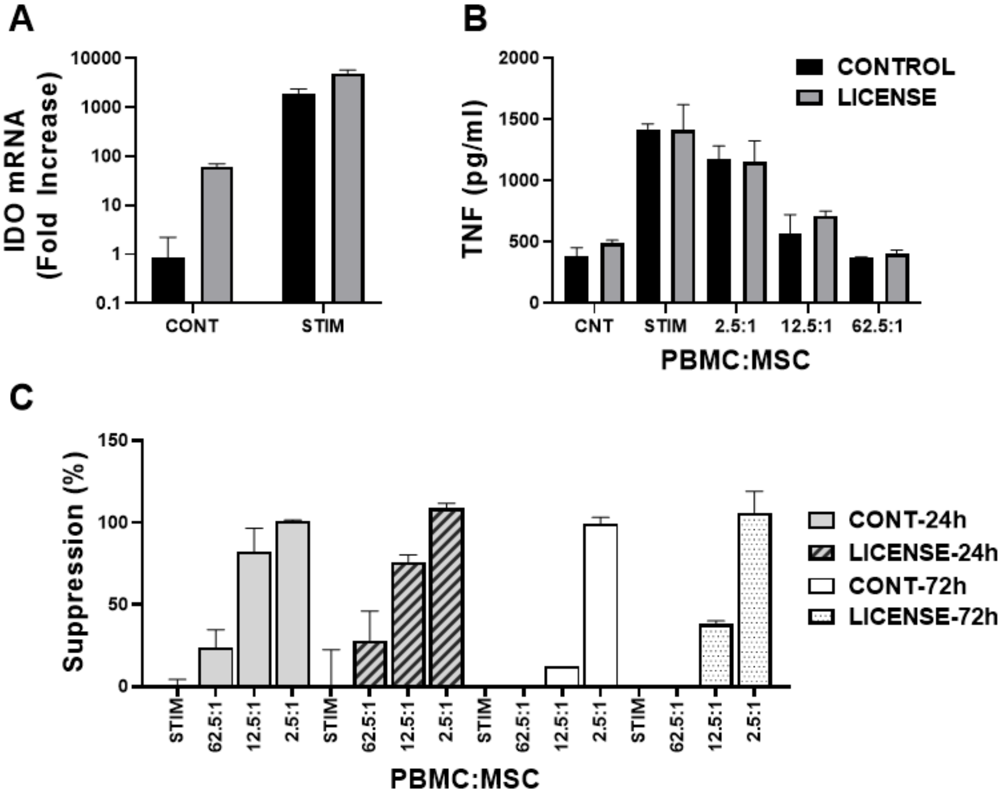

Supplemental Figure 9. Effect of 'licensed' MSCs on hPD PBMC immunosuppression. (A) Validation of licensing- mRNA levels at time 0 for control and licensed BM-MSCs. Data shown is averaged from two experiments on one BM-MSC. Licensing was for 72 hr with 50 ug/ml interferon γ (IFNγ); cells were allowed to attach for 24h prior to hPD PBMC addition with or without 15 ug/ml PHA-P. RT-PCR for target IDO and housekeeping gene B2M were performed. IDO levels of mRNA relative to control, un-licensed BM-MSC are shown. **(B). Control and licensed MSC co-culture effects on TNFα release.** hPD PBMCs were incubated with BM-MSCs allowed to attach for 24h prior to PBMC addition. Ratios of hPD PBMCs to BM-MSCs are listed below the graph. TNFα levels, averages $\pm$ std dev, of duplicate experiments from automated ELISA are shown. (**C). Comparison of suppression of hPD PBMC cytokine release at 24h (Grey and striped bars) or proliferation at 72 h (white and stippled bars) of control (grey and white bars) and licensed (striped and stippled bars) MSC co-culture on hPD PBMCs.** Suppression data is calculated from the same experiments as above.
